# Key regulatory molecules of cartilage destruction in rheumatoid arthritis: an *in vitro *study

**DOI:** 10.1186/ar2358

**Published:** 2008-01-18

**Authors:** Kristin Andreas, Carsten Lübke, Thomas Häupl, Tilo Dehne, Lars Morawietz, Jochen Ringe, Christian Kaps, Michael Sittinger

**Affiliations:** 1 Tissue Engineering Laboratory and Berlin – Brandenburg Center for Regenerative Therapies, Department of Rheumatology, Charité – Universitätsmedizin Berlin, Tucholskystrasse 2, 10117 Berlin, Germany; 2 Tissue Engineering Laboratory, Department of Rheumatology, Charité – Universitätsmedizin Berlin, Tucholskystrasse 2, 10117 Berlin, Germany; 3 Institute for Pathology, Charité – Universitätsmedizin Berlin, Charitéplatz 1, 10117 Berlin, Germany; 4 TransTissueTechnologies GmbH, Tucholskystrasse 2, 10117 Berlin, Germany

## Abstract

**Background:**

Rheumatoid arthritis (RA) is a chronic, inflammatory and systemic autoimmune disease that leads to progressive cartilage destruction. Advances in the treatment of RA-related destruction of cartilage require profound insights into the molecular mechanisms involved in cartilage degradation. Until now, comprehensive data about the molecular RA-related dysfunction of chondrocytes have been limited. Hence, the objective of this study was to establish a standardized *in vitro *model to profile the key regulatory molecules of RA-related destruction of cartilage that are expressed by human chondrocytes.

**Methods:**

Human chondrocytes were cultured three-dimensionally for 14 days in alginate beads and subsequently stimulated for 48 hours with supernatants from SV40 T-antigen immortalized human synovial fibroblasts (SF) derived from a normal donor (NDSF) and from a patient with RA (RASF), respectively. To identify RA-related factors released from SF, supernatants of RASF and NDSF were analyzed with antibody-based protein membrane arrays. Stimulated cartilage-like cultures were used for subsequent gene expression profiling with oligonucleotide microarrays. Affymetrix GeneChip Operating Software and Robust Multi-array Analysis (RMA) were used to identify differentially expressed genes. Expression of selected genes was verified by real-time RT-PCR.

**Results:**

Antibody-based protein membrane arrays of synovial fibroblast supernatants identified RA-related soluble mediators (IL-6, CCL2, CXCL1–3, CXCL8) released from RASF. Genome-wide microarray analysis of RASF-stimulated chondrocytes disclosed a distinct expression profile related to cartilage destruction involving marker genes of inflammation (*adenosine A2A receptor*, *cyclooxygenase-2*), the NF-κB signaling pathway (*toll-like receptor 2*, *spermine synthase*, *receptor-interacting serine-threonine kinase 2*), cytokines/chemokines and receptors (*CXCL1–3*, *CXCL8*, *CCL20*, *CXCR4*, *IL-1β*, *IL-6*), cartilage degradation (*matrix metalloproteinase (MMP)-10*, *MMP-12*) and suppressed matrix synthesis (*cartilage oligomeric matrix protein*, *chondroitin sulfate proteoglycan 2*).

**Conclusion:**

Differential transcriptome profiling of stimulated human chondrocytes revealed a disturbed catabolic–anabolic homeostasis of chondrocyte function and disclosed relevant pharmacological target genes of cartilage destruction. This study provides comprehensive insight into molecular regulatory processes induced in human chondrocytes during RA-related destruction of cartilage. The established model may serve as a human *in vitro *disease model of RA-related destruction of cartilage and may help to elucidate the molecular effects of anti-rheumatic drugs on human chondrocyte gene expression.

## Introduction

Rheumatoid arthritis (RA) is an inflammatory disease characterized by a chronic inflammation of synovial joints that leads to a progressive destruction of articular and periarticular structures, causing severe morbidity and disability [1]. In RA, the extensive infiltration of inflammatory cells into the synovium and the tumor-like proliferation of RA synovial fibroblasts (RASF) cause the formation of a hyperplastic pannus, which aggressively invades and destroys underlying cartilage and bone. Until now, the role of macrophages, T and B cells, neutrophils and RASF in the pathophysiology of RA have been examined extensively [2-6]. Because RASF are known to be one of the key mediators of cartilage destruction in RA [3], comprehensive data have emerged in recent years from gene expression analyses identifying diagnostically and therapeutically highly valued pathophysiological targets of RASF that mediate joint destruction and inflammation [7-9]. Basically, the underlying pathophysiological mechanisms of RASF involve direct cartilage destruction such as infiltration and proteolytic matrix digestion [3,10] and indirect mechanisms triggered by IL-1β and TNF-α, which are secreted from RASF and shift cartilage homeostasis towards catabolism [11]. However, comprehensive data on these indirect effects of RASF mediators on the molecular function of chondrocytes – the single cell type that entirely conducts the cartilage remodeling process – are limited and the underlying molecular pathways still need to be determined thoroughly.

So far, important insights into the mechanisms of RA-related destruction of cartilage have already been obtained from several animal models of arthritis, including destructive arthritis induced by various antigens, transgenic and mutation models and immunodeficient mice [12-16]. In these studies, RA-mediated cartilage destruction was analyzed by histological staining, radiological analysis, and magnetic resonance imaging, which may not reveal the molecular modes of action during cartilage and/or chondrocyte damage in RA. Apart from the challenging molecular examination of cartilage characteristics *in vivo*, the extrapolation of data gained from animal models to the human situation *in vivo *is difficult, thus limiting direct conclusions. Animal models are very complex and cost-intensive systems evoking moral and ethical concerns. According to the '3Rs' concept defined by Russell and Burch in 1959 [17], namely that all efforts to replace, reduce and refine experiments must be undertaken, special attention being given to the development and validation of alternatives (for example *in vitro *models) to animal testing. Tissue engineering offers the opportunity to develop complex physiological *in vitro *models reflecting human significance under well-defined and reproducible conditions. Thus, the objective of the present study was to establish a standardized *in vitro *model to profile the key regulatory molecules expressed by human chondrocytes that are involved in RA-related destruction of cartilage.

Because mature human articular cartilage has a low cell density, expansion of harvested primary chondrocytes was required to obtain sufficient cell numbers, but this led to dedifferentiation of the chondrogenic phenotype. We therefore cultured expanded human articular chondrocytes in alginate beads for 14 days. The alginate bead culture is known to mimic the three-dimensional environment of the cartilage matrix and to preserve the chondrocyte phenotype even in long-term cultures [18]. Furthermore, expanded chondrocytes restore the differentiated phenotype in alginate culture and develop a typical catabolic response to IL-1β after 2 weeks of cultivation, indicating the relevance of the alginate culture to the study of chondrocyte biology on proinflammatory stimulus [19]. Contemporary studies on alginate culture showed that expanded chondrocytes cultured in alginate retain chondrocyte gene expression but the expression level is reduced from the cells' native phenotype; it is therefore not possible to achieve a complete re-differentiation of expanded chondrocytes [20,21]. However, the alginate bead culture was chosen for reasons of standardization; it offers the opportunity (1) to culture expanded chondrocytes batchwise in a phenotype-stabilizing environment, (2) to stimulate chondrocytes batchwise with soluble mediators released from NDSF and RASF, respectively, and (3) to determine the gene expression profile of stimulated chondrocytes by microarray analysis after the isolation of chondrocytes from the alginate.

For reasons of availability, comparability and standardization, human SV40 T-antigen immortalized synovial fibroblasts (SF) derived from a patient with RA (RASF) and from a normal donor (NDSF) were used. Previous studies determined the NDSF cell line to normal healthy synovial fibroblasts that express typical cell surface molecules, maintain the normal expression kinetics of early growth response 1 on stimulation by synovial fluid from patients with RA or by TNF-α and induce the HLA-DR expression in response to interferon-γ [22]. The RASF cell line was determined as a prototype of activated synovial fibroblasts. Genome-wide microarray analysis of RASF compared with NDSF revealed an induced expression of genes associated with the pathomechanism of RA including *IL-1α*, *IL-1β*, *IL-8 *and *CXCL3*, and treatment of RASF with frequently used anti-rheumatic drugs reverted the expression of numerous RA-related genes that were associated with cell growth, metabolism, apoptosis, cell adhesion, and inflammation [23]. Additionally, RASF were shown to synthesize, at the protein level, increased amounts of numerous inflammatory cytokines and matrix-degrading enzymes [23,24].

In brief, our investigation sought to determine the key regulatory molecules of chondrocyte dysfunction that are associated with cartilage destruction in RA. For this purpose, a standardized *in vitro *model of RA-related destruction of cartilage was established. In this model, human chondrocytes were cultured in alginate beads and stimulated with soluble mediators secreted from NDSF and RASF, respectively. Genome-wide differential expression profiling of stimulated chondrocytes was subsequently performed, and expression of selected genes was validated by real-time RT-PCR.

## Materials and methods

### Human chondrocyte isolation and cultivation

The local ethical committee of the Charité Berlin approved this study.

For chondrocyte isolation, human articular chondrocytes from six normal donors *post mortem *without obvious joint defects and macroscopic signs of osteoarthritis were isolated from the medial and lateral condyle of femur bones obtained from the Institute of Pathology at the Charité University Hospital Berlin. The average patient age was 60 years, ranging from 39 to 74 years. Chondrocytes were harvested as described previously [25] and expanded in monolayer culture with RPMI 1640 medium (Biochrom, Berlin, Germany) supplemented with 10% human serum, 100 ng/ml amphotericin B (Biochrom), 100 U/ml penicillin and 100 μg/ml streptomycin (Biochrom). Throughout the experiment, the same pool of human serum (*n *= 5 donors) was used. Medium was changed every 2 to 3 days. Reaching subconfluence, chondrocytes were detached with 0.05% trypsin and 0.02% EDTA (Biochrom) and cryopreserved. After cryopreservation, human chondrocytes were expanded in a monolayer and, after reaching subconfluence again, the cells were trypsinized and subsequently immobilized in alginate beads.

### Cultivation of synovial fibroblasts

Human SV40 T-antigen immortalized SF were derived from a patient with RA (HSE cell line; RASF) and from a normal donor (K4IM cell line; NDSF), respectively. Synovial pannus tissue from a patient with RA was obtained by surgical synovectomy of the knee joint from a patient diagnosed according to the American College of Rheumatology revised criteria as having active RA [26]. Normal donor synovial tissue was obtained during meniscectomy from a 41-year old male suffering from a meniscus lesion [22]. After isolation of the human synovial fibroblasts, the cells were transfected with SV40 TAg expression vector, yielding immortalized synovial fibroblast cell lines [22,26]. Immortalized synovial fibroblasts derived from the patient with RA represent RASF, and immortalized synovial fibroblasts derived from the normal donor patient represent NDSF. SF were expanded in a monolayer with RPMI 1640 medium supplemented with 10% human serum, 100 U/ml penicillin and 100 μg/ml streptomycin. Medium was changed every 2 to 3 days.

### Preparation of alginate bead culture and interactive *in vitro *model

Alginate (Sigma, Taufkirchen, Germany) solution was prepared in 150 mM NaCl and 30 mM HEPES at 3% (w/v) and sterilized by autoclaving. Equal volumes of alginate solution and human articular chondrocyte suspension were combined to yield suspensions with final cell densities of 2 × 10^7 ^cells/ml in 1.5% (w/v) alginate. Spherical beads were created by dispensing droplets of alginate cell suspension from the tip of an 18-gauge needle into a bath of 120 mM CaCl_2_, 10 mM HEPES, 0.01% Tween 80 and 150 mM NaCl followed by gelation for 20 minutes. Beads were cultured in batches in six-well plates for 2 weeks in RPMI 1640 medium supplemented with 10% human serum, 100 ng/ml amphotericin B, 100 U/ml penicillin, 100 μg/ml streptomycin and 170 μM l-ascorbic acid 2-phosphate (Sigma).

**Figure 1:**
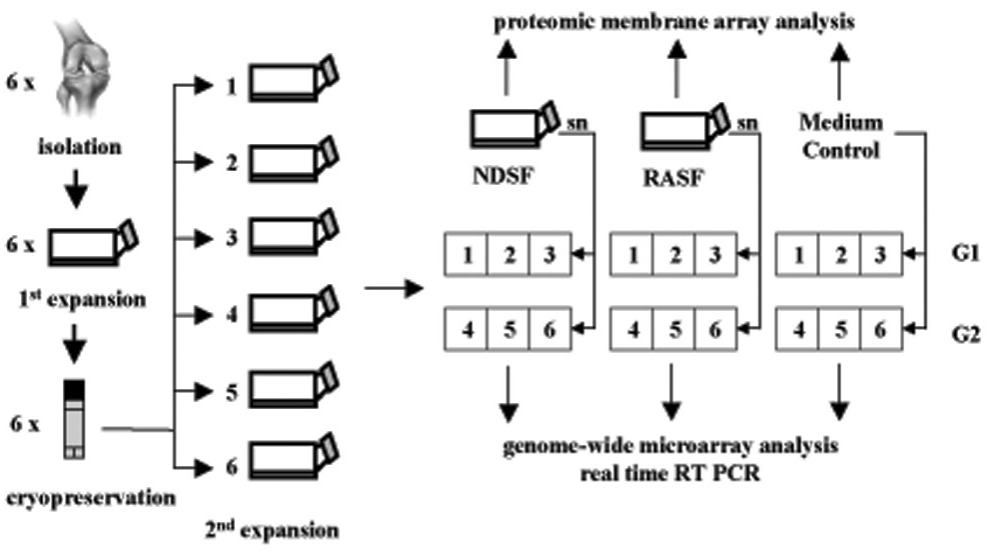
Experimental setup. Human articular chondrocytes were isolated from six normal donors *post mortem *and expanded in monolayer culture. After cryopreservation and a second monolayer expansion, the cells were encapsulated in alginate beads and cultured three-dimensionally for 14 days. Subsequently, the cartilage-like beads were stimulated for 48 hours with supernatants (sn) of SV40 T-antigen immortalized human synovial fibroblasts derived from a healthy, normal donor (NDSF) and from a patient with rheumatoid arthritis (RASF), respectively. Supernatants of RASF (RASFsn) and NDSF (NDSFsn) and medium control were analyzed for soluble mediators with the use of antibody-based protein membrane arrays. Genome-wide expression analyses of NDSFsn-stimulated and RASFsn-stimulated chondrocytes were performed with oligonucleotide microarrays. Additionally, unstimulated chondrocytes were analyzed for baseline expression. Two independent experiments (*n *= 2) were performed for NDSFsn-stimulated and RASFsn-stimulated and unstimulated chondrocytes; each experimental group (G1, G2) consisted of chondrocytes derived from three different donors. Expression of selected differentially expressed genes was validated by real-time RT-PCR.

Medium of NDSF and RASF at 80% confluence was conditioned for 48 hours, and supernatants were adjusted to the same ratio of volume/cell number and stored at -20°C. After 2 weeks of three-dimensional chondrocyte cultivation in alginate beads, medium of cartilage-like beads was replaced by collected supernatants of NDSF (NDSFsn) or RASF (RASFsn). Interactive cultivation was performed for 48 hours (Figure 1). To determine baseline gene expression, a control group of alginate-embedded chondrocytes was treated with cultivation medium for 48 hours.

### RNA purification

Total RNA from stimulated cartilage-like alginate beads was extracted with RNeasy Mini Kit (Qiagen, Hilden, Germany) in accordance with the manufacturer's instructions. Before RNA extraction, alginate beads were solubilized on ice in 55 mM sodium citrate, 30 mM EDTA and 150 mM NaCl, and cells were centrifuged at 800 *g *and 4°C for 5 minutes. Total RNA isolation was conducted in accordance with the manufacturer's protocol. In addition, digestions with proteinase K and DNase I (Qiagen) were performed.

Isolation of total RNA was performed for the six different stimulated donor chondrocytes separately. Afterwards, equal amounts of total RNA from three stimulated donor chondrocytes (1.5 μg from each donor) were pooled, yielding two different experimental groups of NDSFsn-stimulated and RASFsn-stimulated chondrocytes and of unstimulated chondrocytes. From each experimental group, 2.5 μg of combined total RNA was used for microarray applications and 2 μg was used for real-time RT-PCR. Gene expression profiling from pooled RNA samples derived from individual donors with a reasonable replication of pooled arrays has recently been determined to be statistically valid, efficient and cost-effective [27,28].

### Oligonucleotide microarrays

Microarray analyses of RASFsn-stimulated and NDSFsn-stimulated chondrocytes and unstimulated chondrocytes were performed for two experimental groups (*n *= 2). The Human Genome U133A GeneChip (Affymetrix, High Wycombe, UK) that determines the expression level of 18,400 transcripts and variants representing about 14,500 human genes was used for gene expression analysis. Microarray preparation was performed in accordance with the manufacturer's protocol. In brief, equal quantities of high-quality total RNA from experimental groups (2.5 μg of each) were reverse transcribed to single-stranded cDNA. After a second-strand cDNA synthesis, biotin-labeled antisense cRNA was generated by *in vitro *transcription. Next, 15 μg of each generated cRNA preparation was fragmented and hybridized to the oligonucleotide microarray. Washing, staining and scanning were performed automatically with the Affymetrix GeneChip System. Raw expression data were analyzed using (1) GeneChip Operating Software (GCOS) version 1.2 (Affymetrix) in accordance with the manufacturer's recommendations and (2) Robust Multi-array Analysis version 0.4α7 (RMA) [29]. Differentially expressed genes reproducibly showed a fold change of ≤-2 (decrease) or a fold change of ≥2 (increase) as determined by GCOS and RMA data processing. The filtered gene list was functionally annotated with the use of reports from the literature. Hierarchical cluster analysis with signal intensity of differentially expressed genes and the Pearson correlation distance were performed with Genesis 1.7.2 software [30]. Microarray data have been deposited in NCBIs Gene Expression Omnibus (GEO) and are accessible through GEO series accession number GSE10024.

### Real-time RT-PCR

Equal quantities of high-quality total RNA from both experimental groups (2 μg of each) of both NDSFsn-stimulated and RASFsn-stimulated chondrocytes were reverse transcribed with iScript cDNA synthesis kit (Bio-Rad, Munich, Germany) in accordance with the manufacturer's instructions. TaqMan real-time RT-PCR was performed in triplicates in 96-well optical plates on an ABI Prism 7700 Sequence Detection system (Applied Biosystems, Darmstadt, Germany) with Gene Expression Assays for TaqMan probes and primer sets, which were pre-designed and pre-optimized by Applied Biosystems. Quantitative gene expression was analyzed for *chemokine (C-X-C motif) receptor 4 *(*CXCR4*, assay ID Hs00607978_s1), *thioredoxin interacting protein *(*TXNIP*, Hs00197750_m1), *chondroitin sulfate proteoglycan 2 *(*CSPG2*, Hs00171642_m1), *IFN-α inducible protein-6–16 *(*IFI-6–16*, Hs00242571_m1), *cyclooxygenase-2 *(*COX-2*, Hs00153133_m1), *cartilage oligomeric matrix protein *(*COMP*, Hs00164359_m1), *steroid sulfatase *(*STS*, Hs00165853_m1) and *glyceraldehyde-3-phosphate dehydrogenase *(*GAPDH*, Hs99999905_m1). The expression levels of selected differentially expressed genes were normalized to endogenous glyceraldehyde-3-phosphate dehydrogenase expression level and calculated with the 2^-ΔΔ*Ct *^formula (ABI Prism 777 Sequence Detection System User Bulletin no. 2). For statistical analysis, Students' *t*test was applied.

### Proteomic membrane array analysis

The human protein membrane array (RayBiotech, Norcross, GA, USA) simultaneously profiles 30 custom proteins in duplicate. Experiments were performed in accordance with the manufacturer's instructions. In brief, conditioned supernatants of both NDSF and RASF were adjusted with medium to the same ratio of volume/cell number and stored at -20°C. Human cytokine array membranes were incubated for 30 min in 2 ml of blocking buffer and afterwards for 2 hours in 2 ml of sample supernatant at 20°C. After being washed, the membranes were incubated with biotin-conjugated antibodies (1:250 dilution; 1 ml per array membrane) at room temperature for 2 hours and washed again. A solution containing horseradish peroxidase-conjugated streptavidin (1:1,000 dilution; 2 ml) was added and incubation was continued for 2 hours followed by a third washing step. Proteins were detected by enhanced chemiluminescence and the membranes were briefly exposed to X-ray films (Amersham, Munich, Germany) for 30 s, 1 min, 2 min and 4 min. Array images were acquired at a resolution of 300 d.p.i. on a computer photo scanner.

## Results

### Gene expression profiling of stimulated chondrocytes

Because the progressive destruction of articular cartilage is a prominent feature of RA and numerous molecular properties of RASF contributing to cartilage degradation have already been studied, we sought to elucidate cartilage destruction on the basis of chondrocyte gene expression patterns that were induced by soluble mediators secreted from RASF. For this purpose, an *in vitro *model was established that was composed of human articular chondrocytes that had been encapsulated for 2 weeks in alginate beads and then stimulated for 48 hours with supernatant of RASF (RASFsn) or NDSF (NDSFsn).

**Figure 2:**
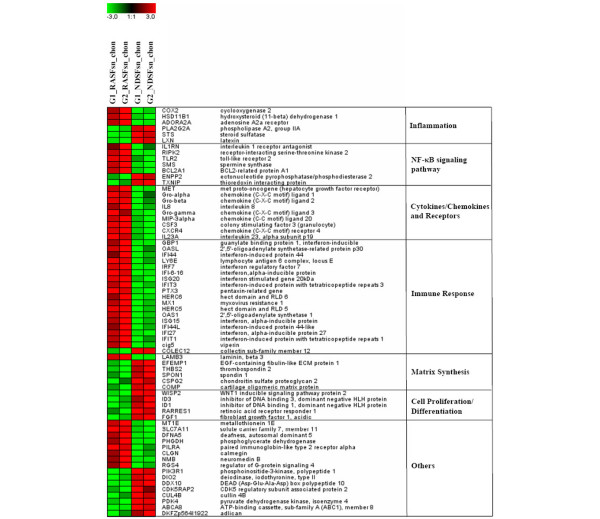
Hierarchical clustering and functional classification of differentially expressed genes. Genome-wide expression analysis was performed for two different experimental groups (G) of chondrocytes stimulated with supernatant of a synovial fibroblast cell line derived from a patient with rheumatoid arthritis (RASFsn) and chondrocytes stimulated with supernatant of a synovial fibroblast cell line derived from normal donor (NDSFsn) (*n *= 2). Each experimental group was a pool of RNA isolated from stimulated chondrocytes that originated from three different donors; that is, group 1 (G1) consisted of equal amounts of RNA from stimulated chondrocytes of donors 1 to 3 and group 2 (G2) of donors 4 to 6. Genes that displayed ≥2-fold increase or ≤-2-fold decrease in RASFsn-stimulated compared with NDSFsn-stimulated chondrocytes determined by both analyses with GeneChip Operating Software and Robust Multi-array Analysis were hierarchically clustered and functionally classified into six groups. Colors represent relative levels of gene expression: bright red indicates the highest level of expression and bright green indicates the lowest level of expression. Expression data from the different experimental groups were compared and showed that the expression patterns were similar for the corresponding experimental groups of both RASFsn-stimulated and NDSFsn-stimulated chondrocytes because they clustered and were therefore most similar to each other, showing little variability.

Alginate beads were generated reproducibly with a spherical shape and a diameter of 2.13 ± 0.13 mm (data not shown). Differential expression analysis of chondrocytes stimulated with RASFsn and NDSFsn was used to determine molecular RA-related patterns of chondrocyte gene expression. GCOS and RMA statistical analyses showed 68 reproducibly differentially expressed genes; 44 genes were induced (fold change ≥ 2) and 24 genes were repressed (fold change ≤ -2). The differentially expressed genes were functionally annotated with reports from the literature and were classified into six functional groups (Table 1). Visualization of these differentially expressed genes by hierarchical clustering demonstrated that the expression patterns of the corresponding experimental groups for both RASFsn-stimulated and NDSFsn-stimulated chondrocytes were similar to each other; corresponding groups clustered and showed little degree of variability (Figure 2).

**Table 1:** Differentially expressed genes in RASFsn-stimulated chondrocytes (FC ≥ 2; FC ≤ -2; RMA and GCOS)

Functional annotation: gene title (gene symbol)	Accession no.	Chondrocyte mean fold change in expression (GCOS and RMA analysis)	Chondrocyte mean signal intensity (GCOS and RMA analysis)		
		
		RASFsn versus NDSFsn stimulation	RASFsn stimulation	NDSFsn stimulation	No stimulation
Inflammation					
Cyclooxygenase-2 (COX-2)	NM_000963.1	2.09	4,474.90	1,793.25	108.4
Hydroxysteroid (11-β) dehydrogenase 1 (HSD11B1)	NM_005525	2.41	2,693.41	955.89	1,263.95
Adenosine A2A receptor (ADORA2A)	NM_000675	4.73	249.59	40.13	27.33
Phospholipase A_2_, group IIA (PLA2G2A)	NM_000300	-2.38	152.32	347.63	787.68
Steroid sulfatase (STS)	AI122754	-3.17	48.36	132.73	412.43
Latexin (LXN)	NM_020169	-5.82	122.35	677.95	610.08
NF-κB signaling pathway					
Interleukin-1 receptor antagonist (IL1RN)	U65590	2.10	1,143.23	278.77	48.83
Receptor-interacting serine/threonine kinase 2 (RIPK2)	AF064824.1	2.12	1,190.60	539.58	22.65
Toll-like receptor 2 (TLR2)	NM_003264	2.25	859.07	322.16	57.30
Spermine synthase (SMS)	NM_004595	2.90	165.10	58.99	40.10
Bcl2-related protein A1 (BCL2A1)	NM_004049	4.90	573.95	94.87	14.63
Ectonucleotide pyrophosphatase/phosphodiesterase 2 (ENPP2)	L35594.1	-3.26	810.00	2,175.34	1,273.98
Thioredoxin interacting protein (TXNIP)	AI439556	-3.50	223.27	622.39	670.70
Cytokines/chemokines and receptors					
Met proto-oncogene (HGF receptor) (MET)	J02958.1	2.02	823.83	333.01	74.13
Chemokine (C-X-C motif) ligand 1 (Groα)	NM_001511.1	2.08	1,414.49	478.28	28.05
Chemokine (C-X-C motif) ligand 2 (Groβ)	M57731.1	2.51	761.47	237.34	10.08
Interleukin 8 (IL8)	AF043337.1	3.16	5,688.87	1,393.65	38.28
Chemokine (C-X-C motif) ligand 3 (Groγ)	NM_002090	3.78	368.84	58.19	16.68
Chemokine (C-C motif) ligand 20 (MIP-3β)	NM_004591.1	5.25	2,028.88	270.12	14.65
Granulocyte colony-stimulating factor 3 (CSF3)	NM_000759	5.61	180.70	51.59	45.18
Chemokine (C-X-C motif) receptor 4 (CXCR4)	AJ224869	5.66	180.71	27.87	16.10
Interleukin-23, α subunit p19 (IL-23A)	NM_016584	11.00	674.98	43.00	39.33
Immune response					
Guanylate binding protein 1, interferon-inducible (GBP1)	BC002666	2.10	450.29	198.92	175.15
2',5'-Oligoadenylate synthetase-related protein p30 (OASL)	AF063612.1	2.38	287.65	98.51	115.65
Interferon-induced protein 44 (IFI44)	NM_006417	2.40	480.29	124.50	238.30
Lymphocyte antigen 6 complex, locus E (LY6E)	NM_002346.1	2.45	569.07	236.97	305.15
Interferon regulatory factor 7 (IRF7)	NM_004030.1	2.48	286.31	93.75	84.93
Interferon-α inducible protein (IFI-6–16)	NM_022873	2.60	550.64	158.67	138.78
Interferon-stimulated gene 20 kDa (ISG20)	U88964	2.61	434.38	153.79	47.20
Interferon-induced protein with tetratricopeptide repeats 3 (IFIT3)	NM_001549	2.69	527.75	137.20	290.03
Pentaxin-related gene, rapidly induced by IL-1β (PTX3)	NM_002852	2.72	340.14	120.25	209.70
Hect domain and RLD 6 (HERC6)	NM_017912.1	3.04	302.12	65.85	117.53
Myxovirus resistance 1, interferon-inducible protein p78 (MX1)	NM_002462	3.09	1,355.03	312.35	557.75
Hect domain and RLD 5 (HERC5)	NM_016323	3.30	608.65	160.90	141.13
2',5'-Oligoadenylate synthetase 1 (OAS1)	NM_002534	4.08	264.81	55.57	70.00
Interferon-α inducible protein, clone IFI-15K (ISG15)	NM_005101.1	4.62	1,943.31	296.19	603.98
Interferon-induced protein 44-like (IFI44L)	NM_006820.1	4.64	691.99	84.63	138.85
Interferon-α inducible protein 27 (IFI27)	NM_005532	5.07	814.69	119.31	154.15
Interferon-induced protein with tetratricopeptide repeats 1 (IFIT1)	NM_001548	5.25	774.38	94.09	361.93
Viperin (cig5)	AI337069	7.14	423.91	34.32	45.65
Collectin sub-family member 12 (COLEC12)	NM_030781	-2.22	648.64	1,347.08	2,518.28
Cell proliferation and differentiation					
WNT1 inducible signaling pathway protein 2 (WISP2)	NM_003881	-2.97	206.25	508.11	4,898.73
Inhibitor of DNA binding 3, dominant negative HLH protein (ID3)	NM_002167.1	-3.74	240.35	715.78	1,465.13
Inhibitor of DNA binding 1, dominant negative HLH protein (ID1)	D13889.1	-4.04	742.79	2,479.99	2,376.33
Retinoic acid receptor responder 1 (RARRES1)	NM_002888	-6.10	115.83	538.71	152.20
Fibroblast growth factor 1, acidic (FGF1)	X59065	-8.51	82.49	513.38	70.23
Matrix synthesis					
Laminin, β3 (LAMB3)	L25541.1	3.05	636.22	196.21	63.28
EGF-containing fibulin-like ECM protein 1 (EFEMP1)	NM_004105	-3.14	170.34	458.87	331.85
Thrombospondin 2 (THBS2)	NM_003247	-3.28	181.13	489.40	483.68
Spondin 1, extracellular matrix protein (SPON1)	AB051390.1	-4.4	56.04	167.4	69.60
Chondroitin sulfate proteoglycan 2 (CSPG2)	NM_004385	-4.53	235.72	670.67	456.13
Cartilage oligomeric matrix protein (COMP)	NM_000095	-5.08	156.77	655.37	308.43
Others					
Metallothionein 1E (MT1E)	BF217861	2.03	1,111.45	554.65	708.38
Solute carrier family 7 member 11 (SLC7A11)	AB040875.1	2.16	692.03	300.52	87.70
Deafness, autosomal dominant 5 (DFNA5)	NM_004403	2.63	1,133.05	379.29	288.20
Phosphoglycerate dehydrogenase (PHGDH)	NM_006623	2.63	171.02	60.04	138.58
Paired immunoglobin-like type 2 receptor α (PILRA)	AJ400843.1	2.82	131.89	32.78	23.75
Calmegin (CLGN)	NM_004362.1	3.20	356.58	87.11	19.30
Neuromedin B (NMB)	NM_021077	3.34	1,163.69	261.33	177.98
Regulator of G-protein signaling 4 (RGS4)	NM_005613.3	3.92	136.81	22.34	42.05
Phosphoinositide-3-kinase, polypeptide 1 (PIK3R1)	AI679268	-3.03	105.59	262.02	182.88
Deiodinase, iodothyronine, type II (DIO2)	U53506.1	-3.10	71.24	193.23	122.88
DEAD (Asp-Glu-Ala-Asp) box polypeptide 10 (DDX10)	NM_004398.2	-3.22	223.36	681.54	252.50
CDK5 regulatory subunit associated protein 2 (CDK5RAP2)	NM_018249	-3.28	250.80	660.54	349.68
Cullin 4B (CUL4B)	AV694732	-3.41	130.55	381.94	83.58
Pyruvate dehydrogenase kinase, isoenzyme 4 (PDK4)	NM_002612.1	-3.65	54.75	179.75	72.75
ATP-binding cassette, sub-family A (ABC1), member 8 (ABCA8)	NM_007168	-3.83	81.80	202.99	141.58
Adlican (DKFZp564I1922)	AF245505.1	-4.90	145.69	486.56	1,835.15

Genes were selected for inclusion if fold change in expression of chondrocytes stimulated with supernatant of a synovial fibroblast cell line derived from a rheumatoid arthritis patient (RASFsn) was ≤-2 (repression) or ≥2 (induction) relative to stimulation with supernatant of a synovial fibroblast cell line derived from a normal donor (NDSFsn) in all specimens (*n *= 2) as verified by GeneChip Operating Software (GCOS) and Robust Multi-array Analysis (RMA) analyses. Gene expression analysis resulted in 68 differentially expressed genes between RASFsn-stimulated and NDSFsn-stimulated chondrocytes: 44 genes were induced and 24 genes were repressed. Differentially expressed genes were functionally categorized into six rheumatoid arthritis-relevant groups and are listed with accession number, mean fold change in expression and mean signal intensity (generated by GCOS and RMA). Annotation of mean signal intensity of RASFsn-stimulated and NDSFsn-stimulated chondrocytes could facilitate the identification of potential rheumatoid arthritis-specific genes for which further investigation may be required. The mean signal intensity of unstimulated chondrocytes is listed for the determination of baseline expression.

Bcl2, B-cell leukemia 2; cig5, cytomegalovirus-inducible gene 5; ECM, extracellular matrix; Gro, growth-related oncogene; HGF, hepatocyte growth factor; HLH, helix–loop–helix; MIP, macrophage inflammatory protein.

Basically, RASFsn-stimulated chondrocytes showed, in comparison with NDSFsn-stimulated chondrocytes, an altered expression of genes associated with inflammation (NF-κB signaling pathway, cytokines/chemokines and receptors, and immune response) and cartilage destruction (matrix metalloproteinases (MMPs), chondrocyte apoptosis, and suppressed matrix synthesis).

As shown in Table 1, genes related to inflammation were differentially expressed in RASFsn-stimulated chondrocytes: *cyclooxygenase-2 *(*COX-2*) and *phospholipase A*_2 _*group IIA *(*PLA2G2A*) regulating the synthesis of prostaglandins, *adenosine A2A receptor *(*ADORA2A*) as an important immuno-modulator of inflammation, and *steroid sulfatase *(*STS*) and *hydroxysteroid *(*11-β*) *dehydrogenase 1 *(*HSD11B1*), which are involved in the biosynthesis of steroid hormones. Moreover, expression of several genes involved in the NF-κB signaling pathway showed differential expression, including *interleukin-1 receptor antagonist *(*IL1RN*), *receptor-interacting serine/threonine kinase 2 *(*RIPK2*), *toll-like receptor 2 *(*TLR2*), *spermine synthase *(*SMS*), *thioredoxin interacting protein *(*TXNIP*) and *BCL2-related protein A1 *(*BCL2A1*). Apart from NF-κB-associated genes, some cytokines/chemokines and receptors were induced, such as *granulocyte colony-stimulating factor 3 *(*CSF3*), *IL-23A *and *hepatocyte growth factor receptor *(*Met*), the chemokines *CXCL1–3 *(*Groα–γ*), *CXCL8 *(*IL-8*) and *CCL20 *(*MIP-3β*), and the chemokine receptor *CXCR4*.

Additionally, profiling of gene expression in RASFsn-stimulated chondrocytes showed a repression of genes involved in cell proliferation and differentiation, and a distinct induction of numerous genes associated with immune response, including *2',5'-oligoadenylate synthetase 1 *(*OAS1*), *2',5'-oligoadenylate synthetase-related protein p30 *(*OASL*) and *IFI-6–16*.

Besides inflammation, RASFsn-stimulated chondrocytes showed a distinct expression of genes associated with cartilage destruction, including chondrocyte apoptosis (*BCL2A1*, *RIPK2 *and *TLR2*) and suppressed extracellular matrix (ECM) synthesis; *cartilage oligomeric matrix protein *(*COMP*), *chondroitin sulfate proteoglycan 2 *(*CSPG2*) *and thrombospondin 2 *(*THBS2*) were repressed in RASFsn-stimulated chondrocytes.

Apart from the 68 differentially expressed genes reaching a fold change of ≥2 or ≤-2, the expression of already established marker genes of cartilage destruction that failed to meet the stringent twofold regulation criteria is listed in Table 2. However, these established RA-related genes showed also differential expression of at least 1.5-fold (GCOS data), including genes involved in oxygen damage and *IL-1β*, *IL-6*, *prostaglandin E synthase *(*PGES*) and genes associated with NF-κB and TNF-α. Moreover, the expression of the matrix-degrading enzymes *MMP10 *and *MMP12 *was induced and the expression of *testican-1 *and genes encoding numerous collagens was repressed.

**Table 2:** Differentially expressed genes in RASFsn-stimulated chondrocytes (FC ≥ 1,5; FC ≤ -1,5; GCOS)

Functional annotation: gene title (gene symbol)	Accession no.	Chondrocyte mean fold change in expression (GCOS analysis)	Chondrocyte mean signal intensity (GCOS analysis)		
		
		RASFsn versus NDSFsn stimulation	RASFsn stimulation	NDSFsn stimulation	No stimulation
Inflammatory/catabolic mediators					
Catalase (CAT)	NM_001752.1	-1.7	672.85	1,221.90	1,386.65
Chemokine (C-C motif) ligand 5 (RANTES)	NM_002985.1	4.1	103.40	25.20	18.95
Chemokine orphan receptor 1 (CMKOR1)	AI817041	2.2	609.45	322.55	89.50
Glutathione peroxidase 3 (GPX3)	AW149846	-1.5	1,083.25	1,617.75	669.90
Interleukin-1β (IL-1β)	M15330	2.2	91.80	34.10	36.45
Interleukin-6 (IL-6)	NM_000600.1	2.6	10,058.00	4,907.15	56.25
Nuclear factor-κB associated gene (NF-κB1)	NM_003998.1	1.5	472.80	312.10	176.75
Nuclear factor-κB associated gene (NF-κB2)	BC002844.1	2.3	125.75	48.25	41.50
Prostaglandin E synthase (PGES)	NM_004878.1	1.9	1,308.70	596.10	123.10
TNF-α-inducible protein 2 (TNFAIP2)	NM_006291.1	2.6	337.65	109.90	98.90
Tumor necrosis factor receptor (TNFRSF1B)	NM_001066.1	2.3	439.20	197.70	67.10
ECM degradation					
Matrix metalloproteinase 10 (MMP10)	NM_002425.1	2.7	587.60	233.90	20.05
Matrix metalloproteinase 12 (MMP12)	NM_002426.1	5.2	161.40	25.90	18.00
ECM formation					
Collagen, type I, α1 (COL1A1)	NM_000088.1	-2.3	472.15	1,182.40	6,603.50
Collagen, type V, α1 (COL5A1)	N30339	-1.9	143.80	296.95	862.60
Collagen, type X, α1 (COL10A1)	X98568	-4.6	36.50	163.90	5.00
Collagen type XI, α1 (COL11A1)	J04177	-1.7	565.80	982.25	1,146.10
Testican-1	NM_004598	-1.8	543.80	1,384.10	2,311.00

Expression levels of rheumatoid arthritis-relevant genes that failed to reach the twofold regulation criteria for both GCOS and RMA statistical analyses are shown. Expression for all listed genes showed a reproducible regulation as determined by GCOS analysis. Genes were functionally categorized into inflammatory/catabolic mediators and genes involved in the degradation and formation of extracellular matrix (ECM), and are listed with accession number, mean fold change in expression (GCOS) and mean signal intensity (GCOS). Mean signal intensity of unstimulated chondrocytes is listed for the determination of baseline expression. The expression was not reproducibly changed for MMPs and collagens that are not listed in this table.

ECM, extracellular matrix; GCOS, GeneChip Operating Software; NDSFsn, supernatant of synovial fibroblast cell line derived from a normal donor; RASFsn, supernatant of synovial fibroblast cell line derived from a patient with rheumatoid arthritis; RMA, Robust Multi-array Analysis; TNFRSF1B, tumor necrosis factor receptor superfamily, member 1B.

Thus, genome-wide microarray data displayed differential expression of distinct genes in human chondrocytes that have already been implicated in inflammatory diseases or cartilage destruction. However, several differentially expressed genes have not yet been described as being regulated in chondrocytes during RA-related destruction of cartilage.

### Validation of gene expression profiles by real-time RT-PCR

The expression profiles of selected genes obtained by microarray analysis were verified by gene expression analysis with real-time RT-PCR. Because numerous RA-relevant genes were differentially expressed in RASFsn-stimulated chondrocytes, representative candidate genes associated with inflammation and cartilage destruction were selected for validation. Among these genes, *COX-2*, *IFI-6–16 *and *STS *were linked with inflammation, and *CSPG2*, *COMP*, *CXCR4 *and *TXNIP *were involved in matrix synthesis and cartilage destruction.

**Figure 3:**
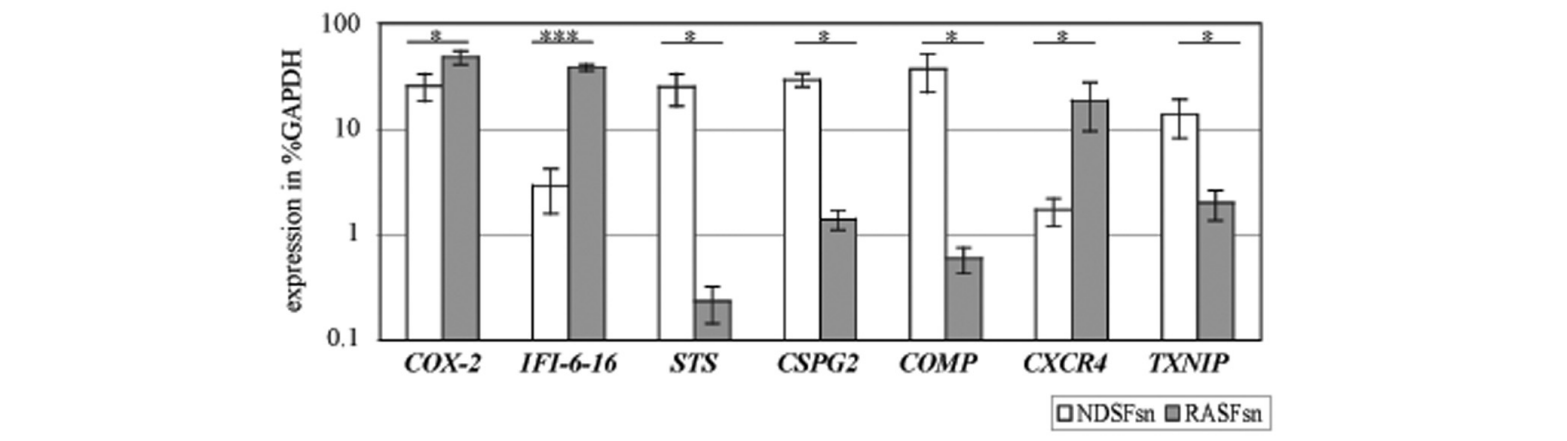
Validation of gene expression of stimulated chondrocytes by real-time RT-PCR. Semi-quantitative real-time RT-PCR of selected genes that were differentially expressed in chondrocytes stimulated with supernatant of a synovial fibroblast cell line derived from a patient with rheumatoid arthritis (RASFsn) as determined by microarray analysis was performed. Real-time RT-PCR gene expression analysis determined that the expression of *cyclooxygenase-2 *(*COX-2*), *interferon-α inducible protein-6–16 *(*IFI-6–16*) and *chemokine (C-X-C motif) receptor 4 *(*CXCR4*) was significantly induced during stimulation of cartilage-like cultures with RASFsn compared with stimulation with supernatant of a synovial fibroblast cell line derived from normal donor (NDSFsn). The gene expression of *steroid sulfatase *(*STS*), *chondroitin sulfate proteoglycan 2 *(*CSPG2*), *cartilage oligomeric matrix protein *(*COMP*) and *thioredoxin interacting protein *(*TXNIP*) was significantly repressed during stimulation with RASFsn. Consistent changes were observed between real-time RT-PCR and microarray analysis for all genes examined. The expression of selected genes was calculated as the percentage of *glyceraldehyde-3-phosphate dehydrogenase *(*GAPDH*) expression. The mean of each triplicate well of both experimental groups is plotted and the error bars represent SD. For statistical analysis, Students t-test was applied (*, *P *≤ 0.05; ***, *P *≤ 0.001).

The expression profiles of *COX-2*, *IFI-6–16 *and *CXCR4 *showed a significant induction, and *STS*, *CSPG2*, *COMP *and *TXNIP *were significantly repressed in RASFsn-stimulated chondrocytes compared with NDSFsn-treated controls (Figure 3), thus confirming the gene expression pattern identified by microarray analysis.

### Protein membrane arrays of synovial fibroblast supernatants

**Figure 4:**
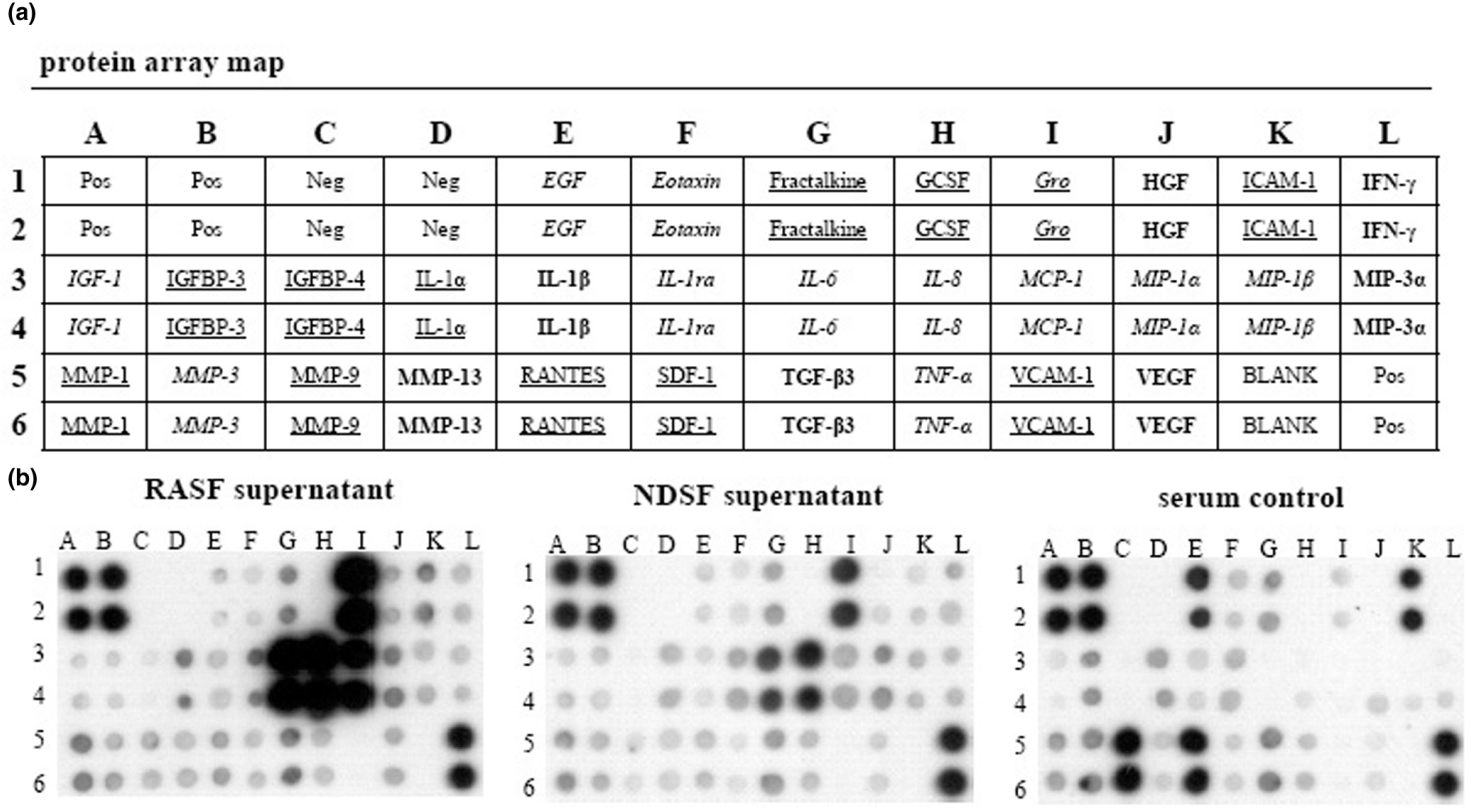
Proteomic membrane analysis of synovial fibroblast supernatants. **(a) **Comprehensive protein membrane array map. The human protein array simultaneously profiles 30 proteins in duplicate, a set of six positive controls and four negative controls. EGF = epidermal growth factor; GCSF = granulocyte colony stimulating factor; Gro = growth-related oncogene; HGF = hepatocyte growth factor; ICAM-1 = intercellular cell adhesion molecule-1; IFN-γ = interferon-γ; IGF-1 = insulin-like growth factor-1; IGFBP-3, 4 = insulin-like growth factor binding protein-3, 4; IL-1α, 1β, 6, 8 = interleukin-1α, 1β, 6, 8; IL-1ra = interleukin-1 receptor antagonist; MCP-1 = monocyte chemoattractant protein-1; MIP-1α, 1β, 3α = macrophage inflammatory protein-1α, 1β, 3α; MMP-1, 3, 9, 13 = matrix metalloproteinase-1, 3, 9, 13; Neg = negative control; pos = positive control; RANTES = regulated on activation, normal T cell expressed and secreted; SDF-1 = stromal cell derived factor-1; TGF-β3 = transforming growth factor-β3; TNF-α = tumor necrosis factor-α; VCAM-1 = vascular cell adhesion molecule-1; VEGF = vascular endothelial growth factor. The sensitivity of antibodies of the RayBio™ human array for the respective proteins differs; proteins in italics: high sensitivity (1–25 pg/ml), in bold: medium sensitivity (100 – 300 pg/ml), underlined: low sensitivity (1,000 – 10,000 pg/ml), Gro determines Groα (low sensitivity) and Groβ (low sensitivity) and Groγ (high sensitivity). **(b) **Supernatants of a synovial fibroblast cell line derived from a patient with rheumatoid arthritis (RASF) and a synovial fibroblast cell line derived from a normal donor (NDSF) were examined for cytokine secretion with the use of antibody-based protein arrays. Because cultivation was performed in medium supplemented with serum, the protein content of the cultivation medium was analyzed as a control. Results are shown after exposure of the array membranes to X-ray films for 2 minutes. The cytokines/chemokines IL-6, CXCL8 (IL-8), monocyte chemoattractant protein-1 (MCP-1), and growth-related oncogene (Gro) showed increased secretion from RASF compared with NDSF and with serum control.

RASFsn-stimulated chondrocytes showed a substantial differential expression of genes that were associated with inflammation and cartilage destruction as determined by microarray analysis and real-time RT-PCR. As shown previously, genome-wide microarray analysis of the respective RASF determined a disease-related expression profile of distinct inflammatory mediators [23]. We therefore hypothesized that soluble mediators were secreted from RASF into the supernatant (RASFsn) and induced the catabolic and inflammatory response of chondrocytes after stimulation. Protein analysis of the supernatant of RASF was used to analyze the secretion of soluble mediators by RASF with the use of custom antibody-based cytokine membrane arrays. A proteomic analysis of these supernatants revealed an increased secretion of cytokines/chemokines by RASF (Figure 4); the secretion of IL-6 (spots G3 and G4), CXCL8 (IL-8; spots H3 and H4), monocyte chemoattractant protein-1 (CCL2/MCP-1; spots I3 and I4) and CXCL1–3 (Gro; spots I1 and I2) was increased compared with NDSF. Because cultivation was performed in medium containing serum, the protein content of cultivation medium supplemented with 10% human serum was analyzed as a control.

## Discussion

To our knowledge, this is the first study that has determined the genome-wide molecular expression pattern of human chondrocytes in response to stimulation with RASFsn and thus provided comprehensive insight into chondrocyte dysfunction during RA-related destruction of cartilage.

RASF are considered to produce a variety of chemokines and catabolic/inflammatory mediators that recruit immune cells to the site of inflammation and facilitate the progressive destruction of articular cartilage [3]; the interaction between both cell types therefore has a fundamental role in RA-related destruction of cartilage. We therefore established an interactive model *in vitro *that determines RA-related molecular processes in chondrocytes induced by soluble mediators that were secreted from RASF. In this model, the chondrocyte alginate bead culture was chosen because it offers the opportunity to culture, in three dimensions, expanded human chondrocytes in a phenotype-stabilizing environment and at the same time to allow interactive culture of chondrocytes and RASF by stimulating cartilage-like cultures with supernatant of RASF (RASFsn). Because direct cell contact between chondrocytes and RASF was not provided, a genome-wide molecular response of chondrocytes to soluble synovial mediators could be determined by microarray analysis.

In previous studies, the respective RASF showed a disease-related expression pattern as determined by genome-wide expression analysis. Moreover, treatment with frequently used anti-rheumatic drugs reverted the expression of numerous RA-related genes [23]; RASF can therefore be considered to be a representative of activated SF. Beyond the RA-relevant expression characteristics, the synovial cell line facilitates standardization, availability and comparability that are appropriate for studies *in vitro*.

In the present study, analysis of protein secretion determined the release of distinct inflammatory mediators; the synthesis of IL-6, CXCL8 (IL-8), CCL2 (MCP-1) and CXCL1–3 (Gro) was increased in RASF compared with NDSF and serum control (Figure 4). This is in line with elevated levels of CXCL8 in supernatants of RASF compared with NDSF as shown by enzyme-linked immunosorbent assay [23]. RASF have already been identified as significant producers of IL-6 and CXCL8. Expression of IL-6 in synovial fluid correlates with markers of inflammation, and blockade of IL-6 signaling is effective in prevention and treatment in models of inflammatory arthritis [31,32]. IL-6 and its soluble receptor have previously been reported to repress important cartilage-specific matrix genes, namely proteoglycans, by means of STAT signaling pathways [33]. In addition, the inflammatory mediators CCL2 and CXCL1–3 have already been identified as being induced in IL-1β-stimulated RASF; CCL2 acting through chondrocyte CCR2 has been described to induce MMP expression and to inhibit proteoglycan synthesis [34].

Strikingly, the amount of inflammatory mediators such as CXCL8 is increased in NDSF supernatant compared with serum control. However, the sensitivity of the protein membrane array is very high for CXCL8, ranging from 1 to 25 pg/ml and it thus detects even very small amounts of protein. The secretion of inflammatory cytokines from NDSF may be due to cultivation of the SF in medium supplemented with human allogenic serum or it may be due to transfection with SV40T. The human serum pool that we used contained detectable amounts of proinflammatory mediators (IL-1β and TNF-α; Figure 4) that may themselves have induced the proinflammatory response in NDSF. Moreover, immortalization with SV40T has been shown to induce the basal expression level of CXCL8 in immortalized SF compared with parental cells [35]. Furthermore, the serum control was characterized by a high content of intercellular cell adhesion molecule-1 (ICAM-1), epidermal growth factor (EGF), CCL5/RANTES and MMP-9 that was, surprisingly, not reflected in SF-conditioned medium. Because all supernatants including the serum control were subjected to the same conditions, the altered protein pattern is most probably due to interaction of the respective proteins with the SF, such as specific binding to cell surface receptors, proteolytic degradation and cell metabolism, or binding of proteins to ECM components on the cellular surface.

An interesting finding was the identification of functional gene groups that are differentially expressed between RASFsn-stimulated and NDSFsn-stimulated chondrocytes (Tables 1 and 2). Gene expression profiling of unstimulated chondrocytes determined the chondrocyte baseline expression of these RA-relevant genes, indicating that SF themselves have an impact on chondrocyte gene expression. However, RASFsn-stimulated chondrocytes are supposed to represent the 'diseased' state, and comparison with the 'healthy' state of NDSFsn-stimulated chondrocytes was used to determine the chondrocyte RA-relevant gene expression pattern independently of SF regulation.

**Figure 5:**
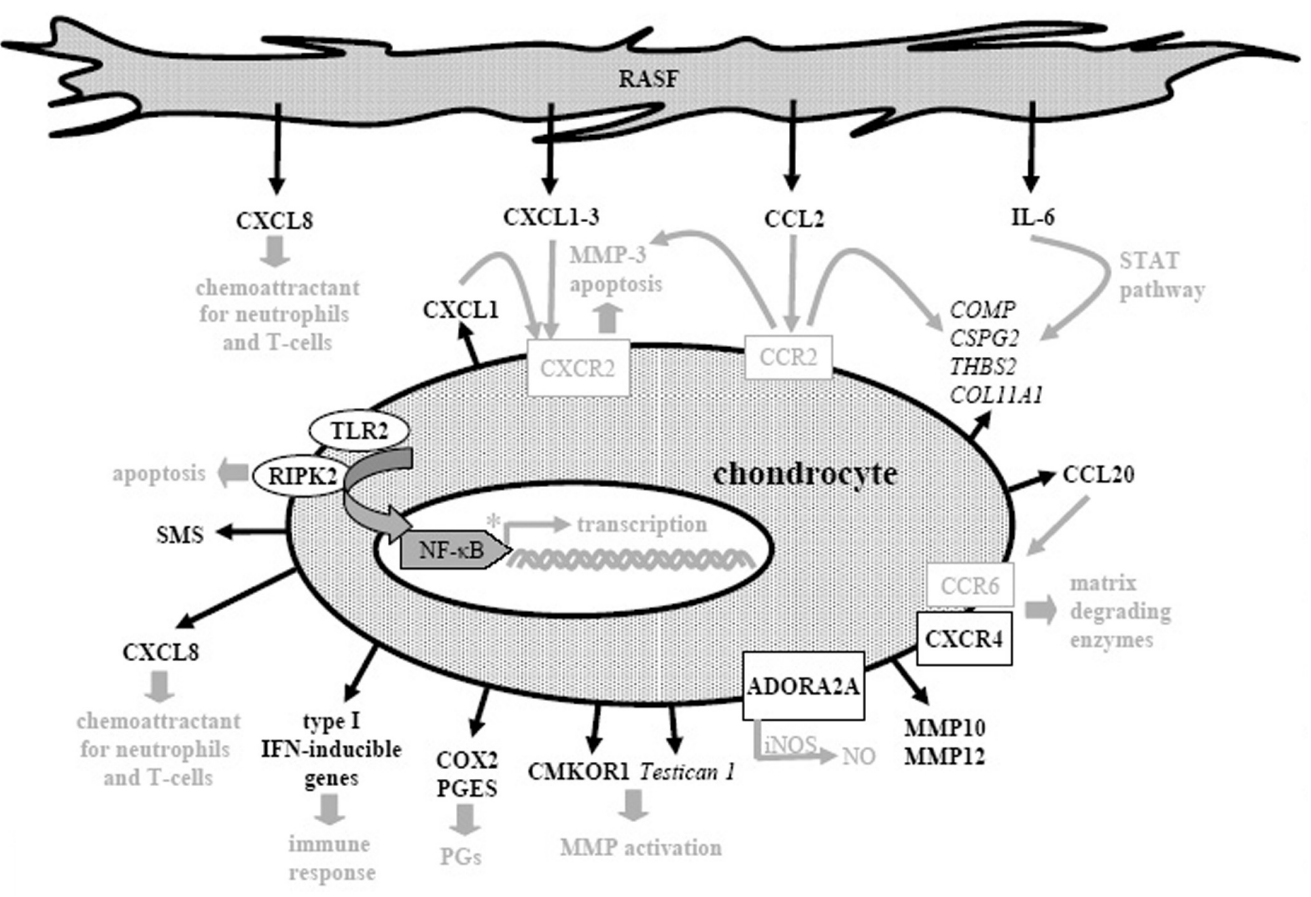
Molecular pathways of rheumatoid arthritis-related cartilage destruction as reflected by the *in vitro *model. Illustration of differentially expressed genes of chondrocytes stimulated with supernatant of synovial fibroblast cell line derived from a patient with rheumatoid arthritis (RASFsn) compared with stimulation with supernatant of synovial fibroblast cell line derived from a normal donor (NDSFsn); induced genes were printed in bold, repressed genes in italics. Genes and text in grey are hypothetical assumptions of the established *in vitro *model for which further validations are still required. Cartilage destruction in rheumatoid arthritis was characterized by a disturbed homeostasis of chondrocyte function that leads to an enhanced cartilage catabolism involving extracellular matrix degradation via matrix metalloproteinases and suppressed extracellular matrix synthesis, induction of catabolic cytokines/chemokines and proinflammatory inducible enzymes, and activation of NF-κB signaling pathway. Thus, the established tissue model provided profound insights into the molecular processes involved in rheumatoid arthritis-related cartilage destruction regarding chondrocyte gene expression patterns. ADORA2A, adenosine A2A receptor; CMKOR, chemokine orphan receptor; COLL11A1, collagen type XI, α1; COMP, cartilage oligomeric matrix protein; COX, cyclooxygenase; CSPG, chondroitin sulfate proteoglycan; iNOS, inducible nitric oxide synthase; MMP, matrix metalloproteinase; NO, nitric oxide; PGs, prostaglandins; PGES, prostaglandin E synthase; RASF, synovial fibroblast cell line derived from patient with RA; RIPK, receptor-interacting serine/threonine kinase; SMS, spermine synthase; STAT, signal transduction and activators of transcription; THBS, thrombospondin; TLR, toll-like receptor; TXNIP, thioredoxin interacting protein.

RASFsn-stimulated chondrocytes showed, in comparison with NDSF stimulation, a regulated expression of genes associated with inflammation (NF-κB signaling, cytokines/chemokines and receptors, immune response) and cartilage destruction (MMPs, chondrocyte apoptosis, suppressed matrix synthesis). Selected differentially expressed genes are illustrated in Figure 5 and printed in black; genes and text in grey are hypothetical assumptions for the established *in vitro *model according to the literature and still require further validation.

Representing the inflammatory aspect, *A2A adenosine receptor *(*ADORA2A*) was induced and is known to be involved in the lipopolysaccharide-induced expression of inducible nitric oxide synthase in chondrocytes, and inducible nitric oxide synthase is a major source of intra-articular production of nitric oxide [36]. Nitric oxide has been described to contribute significantly to chondrocyte death and progressive cartilage destruction by decreasing the synthesis of proteoglycan and collagen type II [37-39], mediating cytokine-dependent susceptibility to oxidant injury [40] and inducing apoptosis [41]. Besides *ADORA2A*, the expression of *COX-2 *as an important pharmacological target gene of inflammation was induced. The formation of prostaglandins by *COX-2 *is a prominent inflammatory process; inhibition of *COX-2 *has cartilage-protective properties, because specific *COX-2 *inhibitors (such as celecoxib) have already facilitated distinct advances in RA therapy [42]. Expression of *PGES*, which is involved in the synthesis of prostaglandin E_2 _downstream of *COX-2*, was induced in RASFsn-stimulated chondrocytes. *PGES *has already been reported to be induced in chondrocytes after proinflammatory stimuli and mechanical stress [43,44]. As shown here, this is consistent with the induction of *COX-2*, *PGES *and *MMP *genes in human chondrocytes cultured in alginate and stimulated with supernatants of RASF.

Furthermore, NF-κB-activating genes were induced in RASFsn-stimulated chondrocytes, including *RIPK2*, *TLR2*, the NF-κB-associated genes *NF-κB1 *and *NF-κB2*, and *SMS*. Promoters of numerous genes involved in inflammation and MMP expression show NF-κB-binding sites [45-47]; NF-κB-dependent genes may therefore be prominent drug targets in RA therapy. RIPK2 has been shown to mediate TNF-α-induced NF-κB activation and induction of apoptosis [48,49].

The induction of numerous cytokines/chemokines fits into the scenario of molecular changes occurring in RA cartilage. Although mature articular cartilage shows little metabolic activity, chondrocytes have previously been described to secrete numerous cytokines/chemokines and chemokine receptors that induce the release of matrix-degrading enzymes and enhance cartilage catabolism [50-52]. RASFsn-stimulated chondrocytes showed an increased expression of *CXCL1–3 *(*growth-related oncogene *α-γ; *Groα-γ*), *CXCL8 *(*IL8*), *CCL20 *(*macrophage inflammatory protein-β*; *MIP-1β*) and the chemokine receptor *CXCR4*. *CXCL1 *has been described to initiate apoptosis in osteoarthritis chondrocytes and induces MMP-3 secretion acting through *CXCR2 *[53]; *CXCL8 *has powerful neutrophil chemotactic properties [54], and *CXCR4 *and *CCL20 *enhance the release of matrix-degrading enzymes [50,55,56]. Inflammatory cytokines that have already been established as markers for RA-related destruction of cartilage, such as IL-1β and IL-6, were differentially expressed in stimulated chondrocytes. Apart from the differential expression of numerous cytokines/chemokines, RASFsn-stimulated chondrocytes showed a decreased expression of genes protecting the cell from oxidative damage (*catalase *and *glutathione peroxidase 3*).

Furthermore, genes directly involved in ECM composition, such as *COMP*, *CSPG2*, numerous *collagens *and *THBS2*, are repressed in RASFsn-stimulated chondrocytes, that characterizes cartilage destruction in RA as a distinct suppression of chondrocyte ECM synthesis. Contributing to RA-related ECM turnover, the expression of *MMP10*, *MMP12 *and *chemokine orphan receptor 1 *(*CMKOR1*) was induced in RASFsn-stimulated chondrocytes. *MMP10 *expression in chondrocytes after cytokine stimulus contributes significantly to collagen breakdown and thus to cartilage degradation [57], and overexpression of *MMP12 *in transgenic rabbits has been shown to facilitate the development of inflammatory arthritis [58]. Treatment of human primary osteoarthritis chondrocytes with *CMKOR1 *agonists has previously been reported to induce matrix degradation and MMP activity, suggesting an important role in the development of osteoarthritis [59]. In addition, *testican 1*, an inhibitor of MMP activation that has been described as having an important role in matrix turnover in osteoarthritis cartilage, was repressed in RASFsn-stimulated chondrocytes [60]. However, neither *CMKOR1 *nor *testican-1 *has yet been described for RA-cartilage turnover.

In summary, our microarray data determined key regulatory molecules of RA-related destruction of cartilage that are consistent with already established marker molecules or that have not yet been determined. As we have established an *in vitro *model that abstracts *in vivo *tissue features, some regulations expected for cartilage destruction, such as a decreased expression of collagen type II or an increased expression of collagenases, were not observed. However, we consider our data to be convincing because the induction of major mediators of inflammation (*COX-2*, *PGES*, *ADORA2A*, *IL-1β*, *IL-6*, *CXCL8 *and *CXCR4*) and cartilage destruction (*MMP10 *and *MMP12*) and the repression of key ECM components (*COMP *and *CSPG2*) are most probably important reasons for chondrocyte dysfunction in RA-related destruction of cartilage.

Because direct cell attachment of SF to chondrocytes was not provided, soluble mediators secreted from RASF regulated the expression of chondrocyte genes and thus disturbed the catabolic–anabolic homeostasis of mature cartilage function. Although the attachment of RASF to cartilage is a significant feature of RA-related destruction of cartilage in comparison with other non-destructive forms of arthritis, direct cell contact between chondrocytes and RASF seems not to be necessarily required for the destructive modulation of chondrocyte function.

## Conclusion

The present study provides a comprehensive insight into the RA-related destruction of cartilage on the basis of chondrocyte gene expression pattern involving marker genes of inflammation and cartilage destruction. We identified molecules already known to be involved in RA-related destruction of cartilage; remarkably, we detected the expression of genes not previously associated with RA chondrocyte dysfunction. Thus, the established *in vitro *model emerged to determine the specific role of distinct genes in the pathogenesis of cartilage destruction in RA and may disclose potent pharmacological targets for cartilage regeneration and repair.

Therefore, this *in vitro *model may help in understanding the molecular effects of anti-rheumatic pharmaceuticals on cartilage regeneration and may facilitate the identification of putative pro-cartilage substances. Because SF treated with frequently used anti-rheumatic drugs showed a reversion of the gene expression of typical RA-related genes [23], a hypothesized drug-related change in the synthesis of disease mediators in RASF may affect the expression in chondrocytes of RA-related target genes of cartilage destruction, demonstrating the molecular effects of anti-rheumatic pharmaceuticals and putative pro-cartilage substances on cartilage regeneration and repair.

## Abbreviations

ADORA2A = adenosine A2A receptor; BCL2A1 = BCL2-related protein A1; CMKOR = chemokine orphan receptor; COMP = cartilage oligomeric matrix protein; COX = cyclooxygenase; CSPG = chondroitin sulfate proteoglycan; ECM = extracellular matrix; GCOS = GeneChip Operating Software; Gro = growth-related oncogene; IFI-6–16 = interferon-α inducible protein-6–16; IL = interleukin; MCP = monocyte chemoattractant protein; MMP = matrix metalloproteinase; NDSF = synovial fibroblast cell line derived from normal donor; NDSFsn = supernatant of NDSF; NF = nuclear factor; OAS1 = 2',5'-oligoadenylate synthetase 1; PGES = prostaglandin E synthase; RA = rheumatoid arthritis; RASF = synovial fibroblast cell line derived from patient with RA; RASFsn = supernatant of RASF; RIPK = receptor-interacting serine/threonine kinase; RMA = Robust Multi-array Analysis; RT-PCR = polymerase chain reaction with reverse transcription; SF = synovial fibroblasts; SMS = spermine synthase; STAT = signal transduction and activators of transcription; STS = steroid sulfatase; THBS = thrombospondin; TLR = toll-like receptor; TNF = tumor necrosis factor; TXNIP = thioredoxin interacting protein.

## Competing interests

CK is an employee of TransTissueTechnologies GmbH (TTT). MS, TH and JR work as consultants for TTT. TTT develops autologous tissue transplants for the regeneration of cartilage and bone. The other authors declare that they have no competing interests.

## Authors' contributions

KA and CL performed the gene expression data processing, participated in the design and coordination of the study and drafted the manuscript. KA, LM and TD conducted the cell culture experiments and performed the protein membrane arrays and the PCR validation studies. TH and JR participated in gene expression data processing and in study design and coordination. CK and MS conceived the study and participated in its design and coordination. All authors read and approved the final manuscript.
